# CT diagnosis of ilioinguinal lymph node metastases in melanoma using radiological characteristics beyond size and asymmetry

**DOI:** 10.1093/bjsopen/zraa005

**Published:** 2021-01-20

**Authors:** M J Wilkinson, H Snow, K Downey, K Thomas, A Riddell, N Francis, D C Strauss, A J Hayes, M J F Smith, C Messiou

**Affiliations:** Department of Academic Surgery, Sarcoma and Melanoma Unit, The Royal Marsden Hospital, London, UK; Department of Academic Surgery, Sarcoma and Melanoma Unit, The Royal Marsden Hospital, London, UK; Department of Radiology, The Royal Marsden Hospital, London, UK; Statistics Department, The Royal Marsden Hospital, London, UK; Department of Radiology, The Royal Marsden Hospital, London, UK; Department of Pathology, The Royal Marsden Hospital (Honorary) and Charing Cross Hospital, London, UK; Department of Academic Surgery, Sarcoma and Melanoma Unit, The Royal Marsden Hospital, London, UK; Department of Academic Surgery, Sarcoma and Melanoma Unit, The Royal Marsden Hospital, London, UK; Division of Radiotherapy and Imaging, The Institute of Cancer Research, London, UK; Department of Academic Surgery, Sarcoma and Melanoma Unit, The Royal Marsden Hospital, London, UK; Department of Radiology, The Royal Marsden Hospital, London, UK; Division of Radiotherapy and Imaging, The Institute of Cancer Research, London, UK

## Abstract

**Background:**

Diagnosis of lymph node (LN) metastasis in melanoma with non-invasive methods is challenging. The aim of this study was to evaluate the diagnostic accuracy of six LN characteristics on CT in detecting melanoma-positive ilioinguinal LN metastases, and to determine whether inguinal LN characteristics can predict pelvic LN involvement.

**Methods:**

This was a single-centre retrospective study of patients with melanoma LN metastases at a tertiary cancer centre between 2008 and 2016. Patients who had preoperative contrast-enhanced CT assessment and ilioinguinal LN dissection were included. CT scans containing significant artefacts obscuring the pelvis were excluded. CT scans were reanalysed for six LN characteristics (extracapsular spread (ECS), minimum axis (MA), absence of fatty hilum (FH), asymmetrical cortical nodule (CAN), abnormal contrast enhancement (ACE) and rounded morphology (RM)) and compared with postoperative histopathological findings.

**Results:**

A total of 90 patients were included. Median age was 58 (range 23–85) years. Eighty-eight patients (98 per cent) had pathology-positive inguinal disease and, of these, 45 (51 per cent) had concurrent pelvic disease. The most common CT characteristics found in pathology-positive inguinal LNs were MA greater than 10 mm (97 per cent), ACE (80 per cent), ECS (38 per cent) and absence of RM (38 per cent). In multivariable analysis, inguinal LN characteristics on CT indicative of pelvic disease were RM (odds ratio (OR) 3.3, 95 per cent c.i. 1.2 to 8.7) and ECS (OR 4.2, 1.6 to 11.3). Cloquet’s node is known to be a poor predictor of pelvic spread. Pelvic LN disease was present in 50 per cent patients, but only 7 per cent had a pathology-positive Cloquet’s node.

**Conclusion:**

Additional CT radiological characteristics, especially ECS and RM, may improve diagnostic accuracy and aid clinical decisions regarding the need for inguinal or ilioinguinal dissection.

## Teaser

The use of non-invasive imaging to detect melanoma lymph node metastases is challenging. This study showed that the use of additional CT morphological characteristics may improve diagnostic accuracy.

Additional features of importance

## Resumen

**Antecedentes:**

El diagnóstico de las metástasis ganglionares (*lymph node*, LN) en el melanoma por métodos no invasivos sigue siendo complicado. El objetivo de este estudio fue evaluar la precisión diagnóstica de seis características de los NL en la tomografía computarizada (*computerised tomography*, CT) para detectar la positividad de las metástasis LN ilioinguinales de melanoma y si las características del NL inguinal podrían predecir la afectación del NL pélvico.

**Métodos:**

Se efectuó un estudio retrospectivo de pacientes con metástasis ganglionares de melanoma atendidos en un solo centro de cáncer terciario entre 2008 y 2016. Se incluyeron pacientes con una evaluación preoperatoria por CT con contraste y disección ganglionar ilioinguinal. Se excluyeron las tomografías computarizadas con artefactos significativos que impidiesen la valoración de la pelvis. En los CT se reevaluaron seis características del NL (ECS: extensión extracapsular, MA: eje mínimo, FH: ausencia de hilio graso, CAN: nódulo cortical asimétrico, ACE: realce anormal del contraste y RM: morfología redondeada) y se compararon con la histopatología postoperatoria.

**Resultados:**

Se incluyeron 90 pacientes. La mediana de edad fue de 58 años (rango 23-85). Ochenta y siete (97%) pacientes tenían enfermedad inguinal con patología positiva y 44 (51%) de ellos presentaban afectación pélvica concomitante. Las características del CT más frecuentes encontradas en los NL inguinales patológicos fueron: MA > 10 mm (97%), ACE (80%), ECS (38%) y RM (38%). En el análisis multivariable, las características de los ganglios inguinales en el CT indicativas de enfermedad pélvica fueron RM (razón de oportunidades, *odds ratio*, OR 3,3; i.c. del 95% 1,2-8,7) y ECS (OR 4,2; i.c. del 95% 1,6-11,3). El ganglio de Cloquet es un mal predictor de diseminación pélvica. En el 50% de los pacientes había metástasis NL en la pelvis, pero solo el 7% tenía un ganglio de Cloquet positivo en el examen la histopatológico.

**Conclusión:**

Algunas características radiológicas en el CT, especialmente la extensión extracapsular y la morfología redondeada, pueden mejorar la precisión diagnóstica y ayudar a las decisiones clínicas con respecto a la necesidad de disección inguinal o ilioinguinal.

## Introduction

Regional lymph node (LN) metastasis is a significant prognostic factor for tumour recurrence and survival in melanoma^1^. Accurate detection of LN metastases is necessary to stage patients correctly, determine surgical treatment strategies, identify patients who may benefit from adjuvant therapy, and may in the future be used to guide neoadjuvant therapy^2^.

 In patients presenting with clinically or radiologically suspected inguinal nodal metastases there are two surgical treatment options: inguinal or ilioinguinal lymph node dissection^3^. These procedures improve local control rates and provide prognostic information. The indications for either operation remain contentious, and they are both associated with a risk of significant morbidity.

In patients with evidence of only inguinal disease, the survival benefit of performing an ilioinguinal dissection over an inguinal dissection alone is unknown. High-level evidence regarding which operation offers most oncological benefit does not yet exist, and is currently being investigated as part of the Evaluation of Groin Lymphadenectomy Extent for Metastatic Melanoma (EAGLE-FM) (NCT02166788) RCT^4^. Under current National Institute for Health and Care Excellence guidance, patients with melanoma AJCC stage IIC (melanoma larger than 4 mm with ulceration) or above (stage III (any lymph node involvement) and stage IV (any metastatic disease)) should have staging CT of the chest, abdomen and pelvis, and MRI of the brain. This has been shown^5^ to change clinical management in up to 20 per cent of patients^5^.

CT is used to exclude metastatic disease and assist in predicting LN disease burden for surgical planning. Currently, size and asymmetry are the most commonly used diagnostic criteria. These are, however, unreliable and may be influenced by multiple factors, such as infection, inflammation, and postoperative changes^6^. If staging investigations presume involvement of pelvic LNs, ilioinguinal dissection is required. Other characteristics that can be identified on CT include extracapsular spread (ECS), absence of a fatty hilum (FH), asymmetrical cortical nodule (CAN), abnormal contrast enhancement (ACE), rounded morphology (RM), and minimum axis (MA). The aim of the present study was to evaluate the diagnostic accuracy of these radiological characteristics in detecting melanoma ilioinguinal LN metastases in patients who had undergone elective ilioinguinal LN block dissection.

## Methods

This was a retrospective single-centre study conducted at the Royal Marsden Hospital, a tertiary oncology centre in London, UK. Patients with melanoma who underwent ilioinguinal block dissection from January 2008 to January 2016 were eligible. Patients without a preoperative staging CT scan of the chest, abdomen and pelvis, and those with CT scans containing significant artefacts obscuring the pelvis, were excluded. CT appearances were reanalysed blindly and compared with final histopathological findings. Further evaluation was performed to investigate whether the CT characteristics within the inguinal LN could be used to predict pelvic LN involvement.

**Fig. 1:**
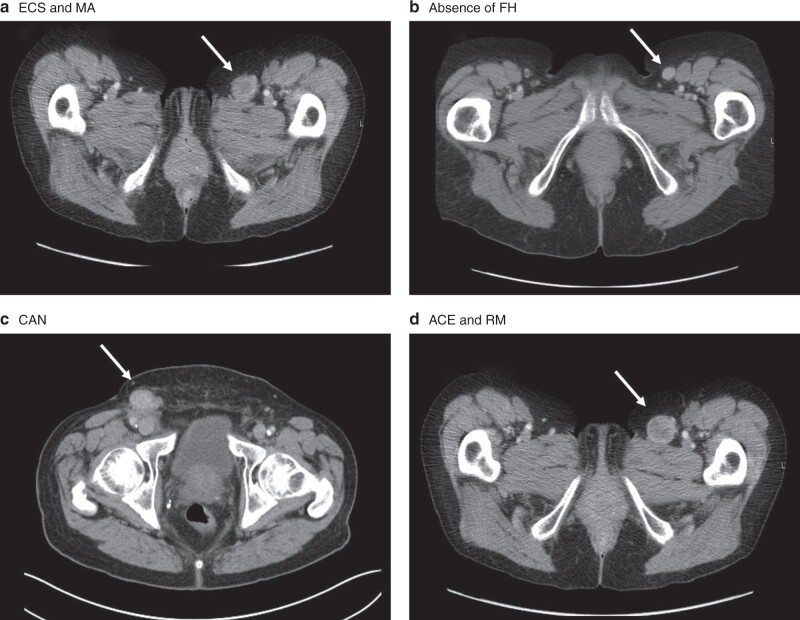
Six CT characteristics of lymph nodes used to indicate presence of lymph node metastases in melanoma **a** Extracapsular spread (ECS) and minimum axis (MA); **b** absence of fatty hilum (FH); **c** asymmetrical cortical nodule (CAN); **d** abnormal contrast enhancement (ACE) and rounded morphology (RM).

Five specified nodal stations, including inguinal, Cloquet’s node, external iliac, pelvic side-wall and common iliac, were each assessed for the following radiological characteristics (*Fig. 1*): ECS (non-uniform enhancement of the capsule or capsular irregularity due to extension of the tumour outside the LN capsule); absence of FH (loss of the normal fat-dense hilum); CAN (disruption of smooth LN cortex by a focal nodule or nodules); ACE (increased enhancement relative to other nodes and skeletal muscle); RM (loss of normal oval shape) and MA (short axis longer than 10 mm).

All CT scans of these patients were reanalysed independently by two experienced consultant radiologists (with more than 10 years’ experience), who were blinded to histopathology results. Discrepancies were resolved by consensus between the radiologists. The diagnostic accuracy of radiology findings was compared with the postoperative histopathology results. At surgery, external iliac and pelvic side-wall (obturator) nodes were resected routinely. Common iliac LNs were removed if thought to be abnormal based on radiological and/or intraoperative findings. All removed LNs were clearly marked by station for pathology review.

### Statistical analysis

On CT, LNs were considered to be positive if any one of the six features (ECS, absence of FH, CAN, ACE, RM and MA) was present. CT was considered to be accurate if CT results (positive or negative) were concordant with the pathology results for each LN station for which pathology results were available. The results were analysed independently by a statistician. For each characteristic, within each nodal station, Fisher’s exact test was performed to test for an association between presence of the characteristic and pathological malignancy, both at the corresponding station and at any pelvic station. Missing values were excluded from the analysis, and no correction was made for multiple comparisons. *P*<0.050 determined statistically significant differences.

Exploratory multivariable analysis was performed to identify the logistic regression model that best predicted pelvic nodal malignancy, using inguinal risk factors as predictive terms. A model with all possible variables (with minimum diameter dichotomized as 10 mm or less and more than 10 mm) was fitted. Backward stepwise methods were used to remove non-significant (at *α* = 0.1) variables.

## Results

Ninety patients were included; 53 per cent were women and 47 per cent were men. Their median age was 58 (range 23–85) years. The median time between CT and surgery was 22 (range 2–115) days, and 96 per cent of patients had surgery within 60 days of CT.

### Lymph node characteristics

Of 90 patients who had ilioinguinal dissection, 88 (98 per cent) had at least one pathological node on histological examination. The two patients without pathological LNs had undergone previous inguinal LN excision biopsy with melanoma-positive nodes, and subsequently had completion ilioinguinal dissection for suspicious pelvic nodes seen on CT. Of the 88 patients with pathology-positive inguinal disease, 45 (51 per cent) had concurrent pelvic disease (17 external iliac, 16 external iliac and pelvic side-wall, 10 pelvic side-wall, and 2 external iliac, pelvic side-wall and common iliac nodes).

The number of LNs resected, CT characteristics, pathology results, and sensitivity, specificity positive predictive value (PPV) and negative predictive value (NPV) by nodal station are shown in *Table 1*.

**Table 1:** Lymph node characteristics of 90 patients with melanoma lymph node metastases determined by histopathological and CT findings according to lymph node station

	Inguinal	Cloquet’s node	External iliac	Pelvic side-wall	Common iliac
**No. of patients with LNs resected**	90 (100)	54 (60)	86 (96)	86 (96)	12 (13)
No. of LNs resected*	9 (2–23)	0 (0–2)	4 (0–14)	5 (0–15)	0 (0–4)
No, of positive LNs*	2 (1–9)	0 (0–1)	1 (1–6)	2 (1–9)	0 (0–2)
**No. of patients with CT-positive LNs**	87 (97)	37 (41)	49 (54)	28 (31)	13 (14)
**No. of patients with pathology-positive LNs**	88 (98)	6 (7)	35 (39)	28 (31)	2 (2)
**CT characteristics in pathological LNs**					
MA >1 cm	85 (97)	4 (67)	31 (89)	16 (57)	2 (100)
ACE	70 (80)	2 (33)	21 (60)	6 (21)	0 (0)
ECS	33 (38)	0 (0)	5 (14)	3 (11)	0 (0)
RM	33 (38)	1 (17)	9 (26)	2 (7)	1 (50)
CAN	13 (15%)	0 (0)	6 (17)	0 (0)	0 (0)
Absence of FH	9 (10)	0 (0)	5 (14)	0 (0)	0 (0)
CT findings†					
PPV (%)	97 (92, 99)	11 (6, 19)	59 (50, 67)	54 (39, 68)	14 (9, 22)
NPV (%)	75 (41, 93)	96 (89, 99)	85 (73, 93)	80 (72, 86)	100
Sensitivity (%)	98 (92, 99)	67 (22, 96)	83 (67, 94)	54 (34, 72)	100 (16, 100)
Specificity (%)	67 (30, 93)	62 (51, 72)	63 (67, 94)	80 (68, 98)	87 (78, 93)

Values in parentheses are percentages unless indicated otherwise; *values are median (range); †values in parentheses are 95 per cent confidence intervals. LN, lymph node; MA, minimum axis; ACE, abnormal contrast enhancement; ECS, extracapsular spread; RM, rounded morphology; CAN, asymmetrical cortical nodule; FH, fatty hilum; PPV, positive predictive value; NPV, negative predictive value.

### Diagnostic accuracy of CT for inguinal and pelvic lymph nodes

In this study, with a high prevalence of LN metastases, 98 per cent of patients had positive inguinal LN on pathology, CT had a sensitivity of 97 (95 per cent c.i. 90 to 99) per cent, specificity of 0 (0 to 84) per cent, PPV of 98 (92 to 100) per cent), and NPV of 0 per cent. The most common CT characteristics in pathology-positive LNs were median axis size greater than 10 mm (97 per cent), ACE (80 per cent), ECS (38 per cent), and RM (38 per cent).

Using the six CT characteristics described above, the diagnostic accuracy of CT for pelvic disease had a sensitivity of 82 (95 per cent c.i. 68 to 92) per cent), specificity of 64 (49 to 78) per cent, PPV of 70 (60 to 78) per cent, and NPV of 78 (65 to 88) per cent. On subanalysis of the separate pelvic LN stations, CT diagnosis of pelvic side-wall LNs was found have a PPV of 54 (39 to 68) per cent and an NPV of 79 (71 to 85) per cent. For external iliac LN stations the PPV was 60 (51 to 69) per cent and the NPV was 80 (74 to 93) per cent), and for common iliac stations PPV was 15 (9 to 24) per cent and NPV was 100 per cent.

### Detection of pelvic lymph node involvement by inguinal lymph node characteristics on CT

Patients diagnosed on CT (owing to the presence of 1 or more positive CT characteristics) as having inguinal LN disease were investigated to evaluate whether this could predict pathology-confirmed pelvic LN disease. CT-positive inguinal disease had a sensitivity of 96 (95 per cent c.i. 85 to 99) per cent and a PPV of 49 (48 to 51) per cent for detecting positive pelvic LNs (specificity 2 per cent and NPV 50 per cent). **I**n multivariable analysis of inguinal LN CT characteristics, variables indicating a higher risk of spread to pelvic LNs were RM (odds ratio (OR) 3.3, 95 per cent c.i. 1.2 to 8.7) and presence of ECS (OR 4.2, 1.6 to 11.3). Patients who were negative for both RM and ECS still had a 27 per cent rate of positive pelvic LN metastasis. The presence of both characteristics, ECS and RM within the inguinal LN correlated with a positive pelvic LN metastasis rate of 80 per cent (*Table 2*).

**Table 2:** Patients with positive pelvic lymph node disease on pathological examination, by inguinal lymph node CT characteristics

	Extracapsular spread	
	No	Yes
**Rounded morphology**		
No	9 of 33	15 of 23
Yes	13 of 22	8 of 10

**Table 3:** Length of the shortest lymph node axis in melanoma-positive and -negative lymph nodes by anatomical lymph node station

Nodal station	Length of lymph node axis according to melanoma involvement (mm)		P*
	Negative	Positive	
Inguinal	8.5 (7–10)	20 (6–54)	0.051
Cloquet’s	20 (4–33)	21 (10–20)	0.641
External iliac	6 (4–17)	11 (6–44)	< 0.001
Pelvic side-wall	5.5 (4–14)	9 (6–27)	0.007
Common iliac	5 (4–14)	10.5 (6–18)	0.087
All nodes	9 (4–33)	17 (6–54)	< 0.001

Values are median (range). *Independent t test.

CT findings were determined to be negative for pelvic LN disease in 35 patients. On pathology review, these were true negatives in 27 patients and false negatives in eight. The presence of ACE (75 per cent), ECS (38 per cent) and RM (38 per cent) was higher in the false-negative group than in the true-negative group (ACE 62 per cent, ECS 20 per cent and RM 14 per cent). The median number of inguinal LN CT characteristics present in the false-negative group was 3 (range 2–5), compared with 2 (1–5) in the true-negative group.

The use of CT to detect melanoma in Cloquet’s node was inaccurate (PPV 11 per cent). This was due mainly to a high false-positive rate for CT, based on median axis length (*Table 3*). Overall, only six of the 90 patients (7 per cent) had a pathologically positive Cloquet’s node. Of the 45 patients with pathology-positive pelvic LNs, 41 did not have a histologically positive Cloquet’s node.

## Discussion

This study evaluated six LN features that are evaluated routinely by radiologists at the authors’ institution to detect LN metastasis in patients with melanoma. Patients with one or more of these CT characteristics in the inguinal LN had a 50 per cent chance of having positive spread of melanoma to the pelvic LN; this increased to 80 per cent in those who had concurrent RM and ECS. Both ECS and RM within the inguinal station LN was predictive of pelvic spread, and thus may be useful in identifying patients who could benefit from ilioinguinal dissection, even in the absence of suspicious CT findings in the pelvis. Absence of all six features correlated with a high likelihood of disease-free pelvic LNs and could guide clinical recommendations for inguinal dissection only.

This study has highlighted the importance of CT LN evaluation beyond the previously used criteria of size and asymmetry. On CT imaging, a normal LN measures less than 10 mm, has a smooth and well defined border, preserved FH, and homogeneous density. Size is the most commonly assessed feature, but is of limited value in differentiating between benign and malignant nodes, and may overlook microscopic disease and partial infiltration^7^. Studies^8^^,^^9^ in other cancers have shown the use of MRI in the assessment of malignant nodes to be equivocal or inferior to that of CT.

The poor reliability of CT and MRI in determining LN metastasis has prompted the evaluation of nodal morphology and the use of dynamic imaging modalities^7^. Previous studies have highlighted the importance of CT in excluding metastasis, but have not shown reliability for diagnosing LN involvement^10^. One study^3^ found that 14 per cent of nodal disease was not detected on CT, with a sensitivity of 58 per cent; however, only the parameters of LN size and asymmetry were used.

Where metastasectomy is planned, [^18^F]fluorodeoxyglucose (FDG)-PET–CT can be used to exclude disease elsewhere, but is not recommended for routine staging^11^. [^18^F]FDG-PET–CT was shown to be 100 per cent sensitive in detecting LNs larger than 10 mm, but only 23 per cent sensitive for nodes smaller than 5 mm^12^. A study^13^ of [^18^F]FDG-PET–CT before sentinel LN biopsy found it could not reliably detect node-positive disease in melanoma, with a high false-negative rate and sensitivity of only 18 per cent. This emphasizes the need for improved imaging criteria based on function and morphology.

Concordant with previous studies^14–16^, Cloquet’s node was not found to be useful in predicting the presence of melanoma metastases in pelvic LNs. Cloquet’s node has a low sensitivity for predicting the pelvic nodal status^16^. CT overdiagnosed Cloquet’s node disease based on size greater than 10 mm, with a PPV of only 11 per cent.

This retrospective study is limited by selection bias. Only patients who had ilioinguinal dissection for suspected pelvic disease were included. Some patients may have had enlarged suspicious nodes on imaging that were then thought to be free from disease on [^18^F]FDG-PET–CT or biopsy. These patients were not included, and this may have had the effect of overstating the predictive performance of the test. Furthermore, the high number of patients (98 per cent) with LN-positive disease limited the ability to determine sensitivity, specificity, PPV and NPV, as the study population did not provide a true representation of disease prevalence. The small numbers of patients in the subgroups of pelvic stations means the results should be interpreted with caution.

## Funding information

National Institute for Health Research

## Notes

Presented to the 16th International Cancer Imaging Society Meeting, Glasgow, UK, October 2016
